# 1697. Safety of Daptomycin in Infants and Children—Six-year Experience from a Pediatric Academic Hospital

**DOI:** 10.1093/ofid/ofad500.1530

**Published:** 2023-11-27

**Authors:** Bryan J Vonasek, Allison M Samuel, Monica C Bogenschutz, Sheryl L Henderson, Jill R Strayer

**Affiliations:** University of Wisconsin School of Medicine and Public Health, Madison, Wisconsin; UW Health, Madison, Wisconsin; UW Health, Madison, Wisconsin; University of Wisconsin School of Medicine and Public Health, Madison, Wisconsin; UW Health, Madison, Wisconsin

## Abstract

**Background:**

Invasive infections caused by gram-positive bacteria are common in pediatric patients and can be challenging to treat. Daptomycin is a lipopeptide antibiotic with activity against gram-positive bacteria and in adults it is a common alternative to first line treatments for complicated skin and soft tissue infections, orthopedic infections, endocarditis, and bacteremia. Data on the safety of daptomycin in the pediatric population, particularly infants and young children, are very limited. The aim of this study was to describe adverse reactions associated with daptomycin use for infants and children.

**Methods:**

This was a retrospective chart review of children < 13 years of age treated with at least one dose of daptomycin while hospitalized at American Family Children’s Hospital (Madison, Wisconsin) between April 2016 and July 2021. Demographic characteristics, comorbidities, indications for daptomycin, culture results, and possible adverse reactions were described using frequencies and proportions for categorical variables.

**Results:**

During the study period, 147 patients received at least one dose of daptomycin, and 72 patients received daptomycin for at least five consecutive days (median: 8.5 days, interquartile range: 6 to 15 days). Although 9.5% reported new-onset loose stools upon daptomycin initiation, all of these instances included other possible contributing factors and were not clearly caused by daptomycin. Elevations in serum creatinine kinase (CK) while on daptomycin were found in six patients, but only two had treatment modifications due to elevated CK: one had daptomycin discontinued, one had daptomycin continued but at a decreased dose. One other patient had daptomycin discontinued specifically due to concern for an adverse reaction of vomiting and irritability associated with drug administration.

**Table 1.**
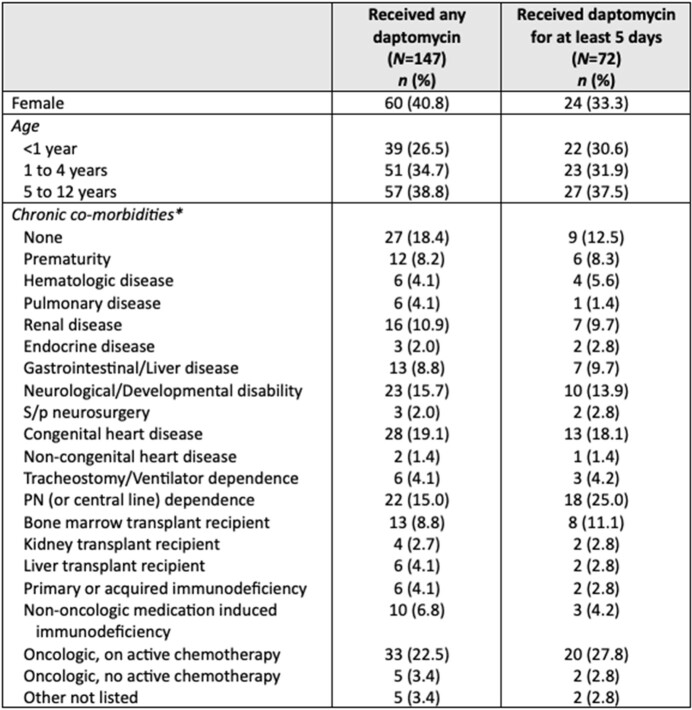
Demographic characteristics and comorbidities of hospitalized children receiving daptomycin, at least one dose or for at least 5 consecutive days. *A particular patient may be enumerated under multiple categories. PN: parenteral nutrition

**Table 2.**
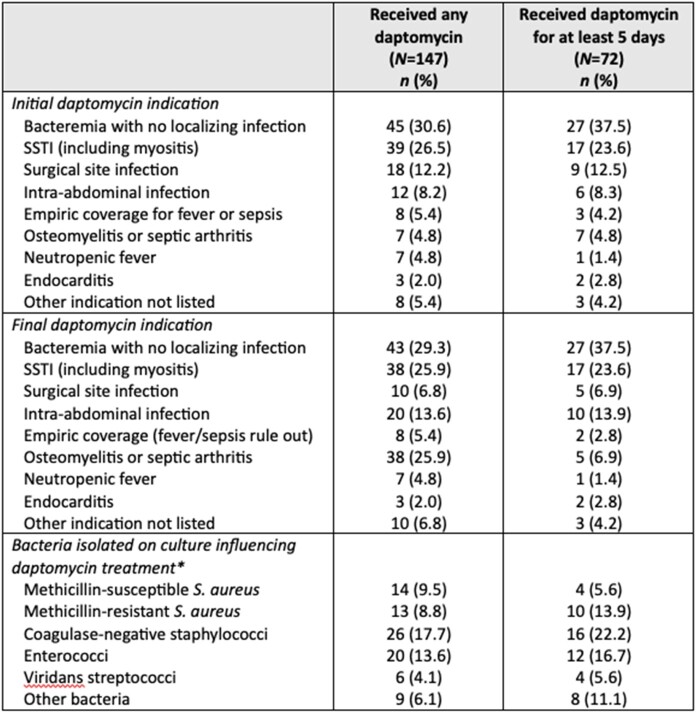
Clinical characteristics of hospitalized children receiving daptomycin, at least one dose or for at least 5 consecutive days. *A particular patient may be enumerated under multiple categories. SSTI: skin and soft tissue infection

**Table 3.**
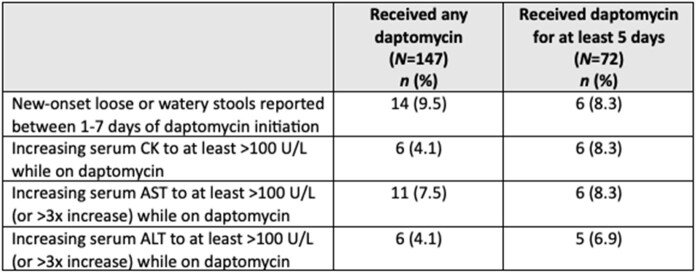
Possible adverse reactions to daptomycin for hospitalized children receiving daptomycin, at least one dose or for at least 5 consecutive days. ALT: alanine transaminase, AST: aspartate aminotransferase

**Conclusion:**

Daptomycin was well tolerated in this pediatric population that included a relatively large number of infants and young children. Only two patients (1.4%) had daptomycin discontinued due to direct concerns for adverse reactions. CK elevations were mild but enough to support current recommendations for regular monitoring of CK while on prolonged daptomycin therapy.

**Disclosures:**

**All Authors**: No reported disclosures

